# Procrastination, Perfectionism, and Other Work-Related Mental Problems: Prevalence, Types, Assessment, and Treatment—A Scoping Review

**DOI:** 10.3389/fpsyt.2021.736776

**Published:** 2021-10-11

**Authors:** Christiane Steinert, Nikolas Heim, Falk Leichsenring

**Affiliations:** ^1^International Psychoanalytic University, Berlin, Germany; ^2^Clinic for Psychosomatics and Psychotherapy, Justus-Liebig-University of Giessen, Giessen, Germany; ^3^Tavistock and Portman NHS Foundation Trust, London, United Kingdom; ^4^Clinic of Psychosomatic Medicine and Psychotherapy, University of Rostock, Rostock, Germany

**Keywords:** work-related problems, procrastination, perfectionism, diagnostics, personality, psychotherapy

## Abstract

Work-related mental problems can be defined as behaviors, emotions and cognitions that impede the successful completion of a task in a given time frame, i. e., the difficulty or inability to achieve important work-related goals. They are highly prevalent but have been neglected in psychology in general and as a target of psychotherapy in particular. Although work-related problems do not represent a mental disorder *per se*, they are associated with severe distress and high psychosocial costs. In this article, the prevalence of work-related problems, associated burden, diagnostic assessment and treatment are reviewed. So far, research has primarily focused on procrastination, i.e., the act of postponing or delaying tasks until the last minute or past the deadline. However, procrastination represents just one type of work-related problems among several others. Further forms of work-related problems are presented (e.g., perfectionism, or work-related problems in the context of specific personality types). The relation of work-related problems to specific mental disorders is discussed. Psychosocial interventions are the treatment of choice for work-related mental problems. However, response rates for the treatment of procrastination are limited, which calls for further research into which treatments work for whom. No evidence-based treatments are currently available for other types of work-related problems, with the exception of perfectionism, a personality trait that is also linked to problems in the field of work. Thus, there is a need to further improve the treatment of work-related problems including procrastination. For other types of work-related problems, effective treatments need to be developed and validated. They may be based on existing manualized treatments and extended by specific aspects or modules focusing on work-related problems.

## Introduction

From a philosophical point of view, meaningful labor is regarded as a fundamental need and characteristic of human beings (1–3). Working is related to both social needs and needs of self-realization (2, 4, 5). Already Freud reportedly considered the ability to love and to work as the cornerstones of mental health (6, 7), which is supported by research showing that even entertaining the thought of unemployment leads to greater mortality-related cognitions (8). Furthermore, the World Health Organization (WHO) acknowledges that work and leisure have a significant impact on health (9). For these reasons, not being able to work effectively and satisfyingly can be regarded as a fundamental psychological impairment and a possible target of psychotherapy.

## Definition

Work-related mental problems is an umbrella term for a heterogeneous group of difficulties that can arise at school, training, university, at work or in creative contexts. They can be defined as behaviors, emotions and cognitions that impede the successful completion of a task in a given time frame, i.e., the difficulty or inability to achieve important work-related goals (10). Research and treatment of work-related problems has primarily focused on procrastination (11, 12), i.e., the act of postponing or delaying tasks until the last minute or past the deadline. Procrastination is consistently related to the aversiveness of tasks and duties, i.e., less pleasant tasks are the ones most frequently associated with procrastination (13). However, procrastination represents just one type of work-related problems among others. A broad spectrum of different types of work-related problems exists, which are described in more detail below (e.g., being afraid of negative evaluation or of making one's own decisions, difficulty continuing work, distractibility, passive-aggressive opposing, or overconfidence). Workaholism and burn-out are not addressed in this review as a recent meta-analysis (14) of 14 samples (*N* = 12,417) showed a strong overlap of depression and burn-out (*r* = 0.80), which casts doubt on whether burn-out depicts a discrete clinical condition. Therefore, burn-out can be understood as a work-related depression and as a psychiatric disorder that will not be subsumed under work-related problems as defined in the present review.

## Methods and Search Terms

In view of the speed at which new research findings are accumulated (15, 16), different forms of evidence synthesis are necessary to inform a particular field about important findings and developments, but also about problems, gaps, limitations and possible future directions (17). This is also true for the field of mental health and psychotherapy. Scoping reviews are particularly useful when a certain field of research or problem is broad or heterogeneous and therefore needs to be outlined and conceptually sharpened, or when a prior comprehensive assessment of findings is missing (17). Thus, scoping reviews include a wider range of literature sources and research designs, serve different purposes and follow different methods in contrast to more well-known forms of reviews such as systematic reviews, meta-analyses or umbrella reviews (16, 18).

Given the broad and exploratory research question, a scoping review (16) was conducted using the English terms “working disorder,” “work disruption,” “inhibition of work,” “procrastination,” “writer's block,” and “perfectionism” in the electronic databases PubMed and PsycINFO. Additionally, the German term for working disorder, respectively, work-related problems (“Arbeitsstörung”) was used. We also searched reference lists of retrieved articles, the psychoanalytic library pep-web as well as books and book chapters on work-related problems (e.g., EBSCO eBook Collection). Additionally, experts in the field were contacted. No time limit was set. Two authors (CS, FL) screened the literature focusing on clinical, etiological, diagnostic and therapeutic aspects. Topics that seemed relevant for this review were chosen by consensus in discussions by all authors. Following the recommendations on scoping reviews by Colquhoun et al. (16), we aimed to synthesize the existing knowledge on work-related problems by “mapping key concepts, types of evidence, and gaps in research” (p. 1294) in a systematic fashion.

## Prevalence and Burden of Work-related Problems

Work-related problems are highly prevalent (11, 19). Procrastination alone, representing just one form of work-related problems, has a prevalence of about 20% (19) with a tendency to increase (11). Furthermore, the majority of university students show high levels of procrastination (11, 20). In general, work-related problems are associated with considerable psychosocial impairments and suffering. Procrastination, for example, is associated with poor academic performance (11, 20), more perceived stress (21) and higher levels of depression and anxiety (21). Procrastination appears in various aspects of peoples' lives, including studying (20), preparing taxes and saving for retirement (11) as well as practicing healthy behaviors (22–24). Thus, it is not restricted to occupational achievements, which emphasizes the great importance of this maladaptive behavior even more.

As work-related problems are not restricted to procrastination, but also include other types described below, the prevalence of work-related problems in general is unknown and can be expected to be considerably higher than that reported for procrastination alone.

Related to this is the general question of when work-related problems should be considered serious impairments in need for treatment (see also sections Work-Related Problems and Mental Disorders and Treatment: Self-Help, Counseling, Psychotherapy, and Evidence). Procrastination, for example, is a behavior that possibly anyone may encounter sooner or later in their life and it is difficult to determine, when such a behavior crosses a “pathological” threshold (25). Personal suffering and failure to achieve important academic or professional goals may be important indicators.

## Work-related Problems and Mental Disorders

Work-related problems do not *per se* represent a mental disorder according to ICD-10, ICD-11 or DSM-5, but may occur in the context of mental disorders. They may have developed on the background of a mental disorder or may belong to the symptoms of a disorder (10). This applies especially to depressive disorders, social anxiety disorder, generalized anxiety disorder, adjustment, trauma- and stress-related disorders, attention and hyperactivity disorder (ADHD), as well as obsessive-compulsive disorder.

*Depressive disorders* are characterized by a loss of interest and reduced energy, sleeping disorders, fatigue, diminished ability to think or concentrate, and a low self-esteem which can severely affect the ability to work effectively. *Social anxiety disorder* and its characteristic fear of being negatively evaluated and ashamed is associated with test anxiety, fear and avoidance of presenting one's work and reduced achievement. *Generalized anxiety disorder* may lead to permanent worries related to one's work. In *adjustment, trauma- and stress-related disorders* emotional distress may be associated with work-related problems. In *ADHD*, hyperactivity, impulsivity, and distractibility may lead to work-related problems. *Obsessive-compulsive disorder* may be associated with an over-emphasized focus on details and perfectionism, compulsive ordering, checking and delaying of tasks. As described below, this applies especially to obsessive-compulsive personality disorder.

“Like sexuality, work is such a major sphere of human activity that any neurosis affects it and nearly any case is, among other things, a neurosis of work.” [(26), p. 203]. Thus, it may be difficult or even impossible to disentangle work disruptions from mental disorders as the former often occur in the context of the latter and work-related symptoms may overlap with symptoms of different diagnostic categories (27). Additionally, they may also be associated with specific personality disorders, personality styles, psychodynamic conflicts, structural impairments or dysfunctional cognitions (see below, Types of work-related problems). In the context of this review we relate to individuals presenting with work-related problems or in whom these problems are especially salient. In a hierarchy of problems, they may be the most urgent to focus on in an attempt to alleviate negative consequences like job loss or academic failure.

## A Psychological Model

Several psychological approaches exist that offer explanations for the etiology and development of work-related problems. From a cognitive-behavioral perspective, a conceptual model has been proposed suggesting an interplay between a person's individual conditions (e.g., skills, dysfunctional cognitions, mental disorders, personality traits, distractibility, aims, motives, resources) and characteristics of the task (e.g., type and amount or number of tasks, interpersonal factors, controllability, safety of job, job image) (10). The higher the degree of discrepancy between individual conditions and task characteristics, the higher the likelihood of work-related problems and work-related discontent (10). Although partly acknowledged by the job image, this model can be extended by the societal dimension. A society putting emphasis on working ethic or professional achievements might mediate the effect of the discrepancy between individual preconditions and task characteristics.

From a psychodynamic perspective, unresolved conflicts (e.g., dependency vs. autonomy, submission vs. dominance, unresolved conflicts with authorities) and ego-structural impairments [e.g., impaired self-regulation including regulation of affects, impulses and self-esteem, impaired judgment and anticipation, disturbed internalized object-relations (28, 29)] may constitute further individual preconditions (30–32). Within the psychodynamic framework, it has been hypothesized that procrastination may be seen as an unconscious rebellion against internalized parents with exaggerated ambitions, whose love depended on achievement (33). When the child's performance does not seem good enough to the parents, the unconscious anger of the child at the parents leads the child to linger and delay tasks. As an adult the same behavior may supposedly bring independence from demanding “parents” as well as deadlines and maybe even time in general (33). Interestingly, empirical research using projective tasks has shown that procrastinating students produced themes related to death more often than punctual students (34). Thus, Blatt and Quinlan (34) conclude that procrastination may be related to an unconscious fear of death, which is dealt with internally through the denial of deadlines, calendars and time. From the point of view of attachment theory (35, 36), the conditional proximity and affection of the caregiver depending on achievements might result in an avoidant attachment pattern. Avoidantly attached individuals might constantly fear that they are not good enough at their job, which in turn might lead to increased anxiety and perfectionism (37). Furthermore, secure attachment correlates with higher work satisfaction (38), whereas insecure attachment styles (avoidant or anxious) are associated with less adaptive coping in response to workplace stress (39).

A more comprehensive overview of models explaining work-related mental problems from behavioral, cognitive, cognitive-behavioral, volitional, third wave and psychodynamic approaches is summarized in Table 1.

**Table 1:** Work-related mental problems from different theoretical and clinical perspectives, with a focus on procrastination.

**Behavioral approach**
Procrastination has been described in behavioral terms as an attempt to balance unpleasant and pleasant experiences (25, 40). In the short-term, procrastination leads to improved mood, which can be seen as a positive reinforcement. In addition, if procrastination serves avoiding unpleasant feelings, it can be seen as negative reinforcement, depending on the alternative tasks and distractions at hand. Interestingly, such distractions may be chosen although they are not *per se* pleasant or even aversive (e.g., cleaning, emptying the dishwasher), as they can be accomplished within a short time frame (10, 25, 40). Behavioral treatments mainly focus on reinforcement, punishment, stimulus control, strategies for time management as well as personalized, meaningful setting of priorities (25, 31, 40).
**Cognitive approach**
This perspective focuses on cognitive processing and attributional theories in relation to work-related actions and their relationship with self-confidence, e.g., many studies showed a relationship between low self-worth and procrastination (11, 25). Anticipating failure may lead to procrastination by actively seeking distracting behaviors as an excuse. When failure occurs, it can be attributed to external adverse circumstances rather than a lack of skill or personal failure (25). Related to this is the cognitive concept of “self-handicapping” (41).
**Cognitive-behavioral approach**
Cognitive-behavioral approaches combine the aforementioned strategies, and may additionally include problem- und motivational analyses, psychoeducation, relaxation therapy, cognitive restructuring, enhancing working techniques, work on interpersonal problems, and finding a more suitable work-life-balance (10, 40–43).
**Volitional approach**
From a volitional perspective, procrastination can be viewed in the context of impaired decision making and disturbed action regulation (25). This may entail problems in evaluating, planning, carrying out, or completing a task and may lead to excessive planning or a lack thereof, vague goals, misjudgment of available time, distraction, lowered self-monitoring or giving up too early. Planning can be become more important than the actual work (25). When deadlines are approaching, enormous efforts can be undertaken which prevents feelings of self-worthlessness but maintains procrastination (25, 40).
**Third wave approach (acceptance and commitment therapy, ACT)**
From the perspective of ACT (44, 45), it is essential to reduce experiential avoidance in favor of psychological acceptance. Experiential avoidance is the process of avoiding unpleasant mental or bodily sensations (46) and takes various forms such as thought suppression, self-harm or procrastination. Psychological acceptance does not mean approval, but the acceptance of the momentary reality (for example of having work-related problems), which is an indispensable precondition for clients in order to assess whether they are able to change it on their own – provided that a change is desirable for them. If change is not possible, again acceptance is required: *That's how it is and I cannot do anything about it* (25). If change is possible, clients are instructed to find out which commitment is required and whether they are willing to invest the necessary time and effort (25). If this is not the case, again acceptance is required: *The problem exists in this way and it will remain because I'm not willing to invest the necessary commitment to make a change*. In order to build up acceptance the therapist and the client should regularly discuss the costs and benefits of neglecting to accept reality, i.e., the consequences of experiential avoidance. Doing so will promote the insight that denying reality is always unfavorable and that acceptance at least offers a possibility to work on undesirable states that can be changed (25). Readiness and motivation for commitment may be achieved by formulating desirable goals. As discussed above, procrastination puts an emphasis on short-term reinforcement by avoiding unpleasant feelings or gaining pleasure. Thus, therapeutic action should constantly and consistently focus on meaningful and desirable long-term goals and foster the commitment to actually put them into practice (25). This may be enhanced by letting clients weigh long-term goals against short-term symptom gains and long-term symptom costs, and checking the functionality of currently applied plans and strategies (25).
**Psychodynamic approach**
In contrast to the aforementioned approaches psychodynamic approaches do not mainly focus on procrastination, but target a wider variety of work-related mental problems. From a psychodynamic perspective, both unresolved conflicts and impaired ego-functions (28, 29) may constitute individual preconditions for work-related problems (30–32). The latter, among others, comprise reality testing, judgment, anticipation, and regulating object relations (28–32). Thus, work ability is closely linked to personality functioning. Within the psychodynamic framework, a central focus lies on (unconscious) inner conflicts, i.e., conflicts including a wish (impulse or affect) and opposing needs, wishes or moral values, which trigger anxiety and must therefore be warded off. It has been hypothesized that procrastination can be seen as an unconscious rebellion against overly ambitious internalized parents (33). From the perspective of attachment theory the conditional proximity and affection of the caregiver depending on achievements might result in an avoidant attachment pattern which in turn might lead to increased fear of not being good enough and perfectionism (35–37). Furthermore, insecure attachment styles (avoidant or anxious) are associated with less adaptive coping in response to workplace stress (39). Perfectionism is another relevant construct regarding work-related problems that is related to psychopathology and maladjustment and has been proposed as a transdiagnostic process of psychopathology (47–50). Especially self-oriented perfectionism may represent a form of counterproductive overstriving (51). Consistent with this broad description of work-related problems, psychodynamic therapy early in history addressed various forms of these problems and related them to personality styles (30–32, 52–54). Psychodynamic treatment strategies focus on conflicts and warded-off affects in the context of a good working alliance. Patients' insight into repetitive (unconscious) conflicts sustaining their problems is improved through confrontation and interpretation. Therefore, transferential and countertransferential aspects and the way in which patients “work” in therapy are focused, e.g., getting distracted or doing the work for the therapist by offering explanations. When impairments in ego-functions are dominant, more supportive techniques to build or restore ego-functions are applied (55).

## Types of Work-related Problems

As noted above, research and treatment has primarily focused on procrastination (11). However, there exists a broad spectrum of different types of work-related problems. Perfectionism is another relevant construct in this area and is consistently related to psychopathology and maladjustment (47), which is why it has been proposed as a transdiagnostic process of psychopathology (48). In professors of psychology, for example, self-oriented perfectionism (i.e., demanding perfection of oneself) was negatively related to the total number of publications, number of first-authored publications, number of citations, and journal impact rating, even after controlling for competing predictors (e.g., conscientiousness) (51). The authors concluded that self-oriented perfectionism may represent a form of counterproductive overstriving that limits research productivity (51). Whereas earlier research regarded procrastination as closely related to perfectionism, the relationship has turned out to be more complex (56, 57). Perfectionistic concerns were found to be positively related to procrastination, whereas for perfectionistic strivings a negative relation to procrastination was reported (57).

Perfectionism was found to be multidimensional, including self-oriented perfectionism (high standards on one's own performance), socially prescribed perfectionism (experiencing others as demanding perfectionism), other-oriented perfectionism (high standards on others' performance) and perfectionistic concerns vs. perfectionistic strivings (58).

Perfectionistic concerns were found to be substantially and positively related to neuroticism whereas perfectionistic strivings were found to be substantially and positively related to conscientiousness (59). As procrastination, perfectionism seems to increase over time (60). Furthermore, maladaptive perfectionism was found to be an important personality trait in imposter syndrome (48, 61), which is characterized by low self-esteem, fear of being exposed and over preparing. Counter-intuitively, individuals with imposter syndrome, who are typically described as delivering superior work, score low on conscientiousness (61, 62), which questions this public image and thus could be classified as work-related problem. Related to this, a combination of perfectionism, low self-esteem and the fear of being exposed may underly the phenomena of feelings of incompetence as described in novice psychotherapists (63, 64), which might prevent them from working effectively with patients or supervisors.

Furthermore, if the various personality types are too pronounced or personality disorders (65) are present, they may be related to specific forms of work-related problems (10, 32). On the other hand, certain personality styles (66) are associated with potential strengths which may be focused and fostered in psychotherapy or counseling. Other personality styles, which are commonly regarded to be maladaptive or psychopathological might be considered a strength in certain work contexts. For example, senior business managers show significant elements of personality disorders, particularly of psychopathic personality traits (67). Related to this is a line of research on the so called “dark triad” (68, 69), including the traits machiavellism, narcissism and (subclinical) psychopathy and covering counter-productive as well as advantageous aspects of these traits when it comes to the workplace and attaining leading positions (69–71). According to a review on the dark triad [(69), p. 206], Hogan coined the term that people showing higher levels of dark traits may “get ahead of” but not necessarily “along with” others in working environments (72).

Following König (32) and Fydrich (10) individuals with an ***anxious-avoidant*** ***personality disorder/interactional style*** may be permanently worried about their performance and reluctant to take any personal risk or engage in new activities including work. On the other hand, they may be able to anticipate problems and question premature actions and decisions. Individuals with a ***dependent interactional style*** tend to avoid making independent decisions, are in an excessive need of reassurance from others, and have difficulties initiating projects. As a more positive aspect, they may make good deputies, balancing out interpersonal difficulties among colleagues. A ***compulsive style*** may be related to over-emphasizing details while losing track of what is important, dysfunctional perfectionism, reluctance to delegate tasks and to postponing activities. On a positive note, a strength of this style may be a particularly thorough working style, relieving colleagues with different working styles from bothersome duties. A ***histrionic interactional style*** is associated with an initial excitement when beginning a new task, followed by problems in sticking with the task when it becomes less exciting or involves routine activities. A focus on attention seeking, physical appearance, sexually provocative or charming behavior may be related to success in some areas of work but lead to problems and frustration in others. Individuals with strong ***narcissistic tendencies*** tend to overestimate their abilities, exaggerate their achievements and talents, have an excessive need for admiration and a sense of entitlement. They may expect to be recognized as superior without commensurate achievements or feel entitled to a particularly favorable treatment while being reluctant to take over simple or routine tasks that are perceived as unreasonable (10, 32). On the other hand, they may be very successful, e.g., if they are able to reflect on some of their socially problematic styles.

Consistent with this description of work-related problems linked to personality traits or disorders, psychodynamic therapy early in history addressed various forms of work-related problems and related them to personality traits (30–32, 52–54), including those described for the personality disorders listed above (10). Furthermore, they discuss additional personality styles, such as the schizoid style (e.g., being able to see the whole picture but neglecting details, or avoiding the exchange with colleagues which may put constraints on their career), the depressive style (e.g., difficulties in starting a work or setting priorities) (32) or fear of success (52, 53, 73, 74). Fear of success is related to the assumed consequences of success (e.g., surpassing important others) (52, 53, 73, 74). Whether fear of success is particularly prevalent in women is not yet clear (74). Psychodynamic models of work-related problems focus on associated personality traits, unresolved conflicts and impairments in ego-functions (28) (e.g., self-regulation of affects, impulses and self-esteem) (30–32, 52, 53). On a conscious level, test anxiety, for example, represents the fear of not being successful, but may unconsciously represent the opposite, that is the fear of being successful and being more successful than important others such as one's mother or father which may trigger feelings of guilt.

The ability to work entails adequately handling demands of the external world and is therefore closely related to ego-functions (28). Ego functions in a work-related context enable a person to differentiate between fantasy and reality (reality testing), make reasonable decisions (judgment, anticipation), deal with interpersonal difficulties (regulation of object relations), regulate self-esteem and emotions (self-regulation), set oneself apart from inner or outer stimuli (stimulus barrier), concentrate (autonomous functioning) or integrate contradicting tasks (e.g., being in charge at home but mostly follow instructions at work (synthetic-integrative functioning) (28). Thus, work ability is closely linked to personality functioning and it can be expected that more severe impairments in functioning lead to more severe difficulties in a broad range of areas (e.g., in borderline or narcissistic personality pathology or psychosis).

Work-related problems may be ego-syntonic or ego-dystonic. As long as individuals are not confronted with work-related failures or interpersonal problems, their working style can often be expected to be ego-syntonic. For example, as long as someone with a narcissistic style is successful, it is rather the others who are likely to suffer from this personality style, e.g., the employees of a narcissistic boss. Patients with an anxious or dependent style may function well as long as there are people they can rely on (directing objects). Thus, there are specific situations that may trigger failures at work, such as the loss of a person to rely on in individuals with an anxious or dependent style. An obsessive-compulsive style may be functional as long as the subject is not procrastinating or lost in details. The same is true for a histrionic, schizoid or depressive style as long as attention seeking, neglecting details or problems in setting priorities, respectively, do not result in work-related failures. Work-related failures and related interpersonal difficulties may contribute to work-related problems becoming ego-dystonic which can be a helpful first step toward insight into problems and seeking help or treatment.

Another psychological phenomenon in the context of work that occurs in professional and creative writing, but is sometimes [maybe unjustifiably (75)] also used in a student context, is “writer's block,” a term coined by Bergler in the mid 20th century (76). Writer's block can be defined “as an inability to begin or continue writing for reasons other than a lack of basic skill or commitment” [(77), p. 18]. Hereby, “blocking is not simply measured by the passage of time (…) but by the passage of time with limited productive involvement in the writing task” [(77), p. 18]. Writer's block can be associated with feelings of anxiety, anger, or confusion and can take different forms, i.e., producing too little text or producing only fragments (77). Furthermore, it has been described as being related to perfectionism and procrastination (78). A famous and funny series of articles around this phenomenon concerns the unsuccessful self-treatment of writer's block [e.g., (79–81)]. Different causes have been attributed to writer's block among them the lack of basic writing skills or strategies (77), fear of imperfection (78, 82), inner conflicts around expectations (75, 76, 83) or the loss of the ability to be spontaneous or playful (75, 84). An important aspect in writer's block seems to be related to achievement and success. As Amado put it based on ideas originally presented by Freud [“On those wrecked by success” (52)]: “The elimination of an external frustration, the achievement of a wish, uncovers the internal frustration in all its intensity. This is the very Freudian idea that sometimes the last thing you want is to get what you want” [(75), p. 2]. This may be related to creative writing but also be true in academic writing and other work-related contexts when important goals have been completed and certain positions have been reached.

## Work-related Problems From a Dimensional View

With its alternative “hybrid” model of personality disorders (AMPD), the DSM-5 provided a more dimensional view of personality disorders that is compatible with the specific work-related problems discussed above for the various types of personality disorders. The criteria of disturbances of self (including the subdimensions identity and self-direction) and interpersonal relationships (including the subdimensions empathy and intimacy), which are central to the DSM-5 alternative model of personality disorders [(65), p. 762], encompass features or mechanisms of work-related problems. This applies, for example, to avoidant personality disorder for which DSM-5 lists low self-esteem and shame (identity), unrealistic standards associated with reluctance to pursue goals or take personal risks (self-direction) and sensitivity to criticism (empathy) [(65), p. 765], which may contribute to work-related problems. For narcissistic personality disorder [(65), p. 767], this applies, for example, to exaggerated self-appraisal (identity), setting goals on gaining approval, unreasonably high personal standards (self-direction), impaired ability to recognize the needs of others (empathy), and relationships serving self-esteem regulation (intimacy). In obsessive-compulsive personality disorder [(65), p. 768], work-related problems are associated with a sense of self that is predominantly derived from work (identity), difficulties in completing tasks, and unreasonably high and inflexible internal standards (self-direction). Furthermore, work-related problems in obsessive-compulsive personality are characterized by difficulties in understanding and appreciating the ideas and feelings of others (empathy) as well as rigidity and stubbornness that negatively affects relationships with others (intimacy). In antisocial personality disorder, traits associated with work-related problems are egocentrism (identity), goal-setting based on personal gratification, lack of prosocial internal standards (self-direction) and concerns for feelings or suffering of others (empathy) as well as exploitation as a primary means of relating to others (intimacy). In borderline personality disorder [(65), p. 766], work-related problems may be associated with an unstable self-image (identity), instability in goals (self-direction), compromised ability to recognize the feelings of others associated with interpersonal hypersensitivity (empathy), intense unstable and conflicted close relationships that are viewed in extremes of a idealization and devaluation (intimacy).

The categorical diagnoses of personality disorders are not retained in the latest version of the ICD (ICD-11). Instead, with the exception of borderline personality, existing distinct categories are replaced by a dimensional model which is built on a psychodynamic model of personality functioning and determines the severity of the personality pathology (i.e., mild, moderate, severe), based on functioning of aspects of the self and interpersonal dysfunction as well as six trait domain qualifiers whose use is optional (85). The latter comprise negative affectivity, detachment, disinhibition, dissociality, anankastia and a borderline pattern qualifier. A “tentative cross walk” from ICD-10 personality disorders to ICD-11 trait domain qualifiers has been provided [(85), p. 8]. For example, in terms of ICD-11 the previous dependent personality disorder consists of the traits negative affectivity (with specific trait features like low self-confidence and anxiety) and low dissociality (in the sense of an overly consideration of others' needs) while narcissistic personality disorder can be described in terms of high dissociality (here in the sense of grandiosity and entitlement) and negative affectivity (with the specific trait feature of dysregulated self-esteem). Thus, with regard to DSM-5 and ICD-11 it is important to note that work-related problems may be conceptualized in the context of dimensional approaches to personality disorders which will become more important in the future (86).

## Diagnostic Assessment

As other psychiatric symptoms, work-related problems are not an isolated phenomenon but must be considered in the context of a person's life. For this reason, a thorough biographic interview is required. Comorbid mental disorders need to be assessed, for example, through structured interviews such as SCID-5-CV (87) and SCID-5-PD (88). To assess the level of personality functioning and of specific ego-functions, the operationalized psychodynamic diagnosis (OPD), the AMPD described in DSM-5, the diagnostic assessment according to ICD-11 (85) or the structured interview of personality organization [STIPO, (89)] may be applied (29, 65). Interestingly, the AMPD and related diagnostic instruments emphasize the importance of “love and work as the cornerstone of humanness” [(90), p. 318, (91)]. The STIPO, for example, covers several domains (i.e., identity, object relations, defenses, aggression and moral values), focusing on the past 5 years. The very first section of the STIPO deals with identity and the capacity to invest. Its items are centered around work effectiveness, work ambition, and work satisfaction {e.g., how effective someone is in their work and how important work is, whether the work performance is in accordance with once abilities and whether working is enjoyed [(92), p. 4–6]}. With regard to interpersonal relationships, conflicts with co-workers, supervisors, bosses, subordinates etc. are explored [(92), p. 25]. Additionally, perfectionistic strivings and the fear of being negatively evaluated by others when things are not perfectly right are investigated {higher level defenses [(92), p. 46]}. Thus, this may be a good starting point for a more in depth exploration in people presenting with work-related problems.

In addition, especially in psychodynamically oriented treatments, dreams and phantasies about work, exploration of biographical and familial attitudes toward work and the therapist's countertransference should be explored. The idea behind using the countertransference for diagnostic purposes is that work-related disorders entail problems in internal and external object relationships that can be understood and dealt with in the context of the therapeutic relationship. The above-mentioned psychodynamic model conceptualizes work-related problems as being inherently interpersonal, i.e., embedded in real, internalized or imagined object-relations, which can be assessed by the Core Conflictual Relationship Theme Method (CCRT) (93). Based on relationship anecdotes, the CCRT method operationalizes central relationship patterns by systemizing wishes, needs and motivations of a subject (W), the anticipated response of others to the subject's wishes (RO) and the following response of the subject (RS) to the anticipated response of others to their wish. For example, the wish for appreciation of achievements (W) might be associated with the experience that caregivers react with withdrawal of affection or hostility (RO) resulting in being submissive or even fearing success (RS). A student with this CRRT might suffer from procrastination as success (W) is unconsciously associated with losing the affection of important others (RO) and thus own achievements are sabotaged (RS).

Most diagnostic instruments for the assessment of work-related problems refer to procrastination [e.g., General Procrastination Scale, Adult Inventory of Procrastination, Decisional Procrastination Scale, Adult Inventory of Procrastination Scale, Irrational Procrastination Scale, Pure Procrastination Scale (42, 94–97)]. For the assessment of perfectionism, several self-report instruments are available such as the Multidimensional Perfectionism Scale, the Perfectionistic Self-Presentation Scale or the Perfectionism Cognitions Inventory (47, 98, 99). Additionally, a 58-item list of work-related problems in the context of academia has been developed, focusing on different problems like procrastination, exam anxiety, writer's block, lack of motivation, tiredness, demands, and worries about the future (27). The diagnostic assessment of other forms of work-related problems seems to have been neglected. Especially instruments focusing on work-related problems in the context of personality are lacking. For this reason, the authors of this review are currently developing a self-report questionnaire comprising items on work-related problems in the context of different personality styles (unpublished). The questionnaire is currently being empirically examined in a sample of adults and will be psychometrically evaluated, improved, and published.

## Treatment: Self-help, Counseling, Psychotherapy, and Evidence

Work-related problems may be addressed by self-help programs, counseling or psychotherapy.

According to some authors, clinical experience and research evidence show that problems related to procrastination cannot simply be addressed by advice on better time management or by putting together to-do-lists (13, 40). Thus, more deeply rooted dysfunctional aspects have to be targeted in those suffering from more severe work-related problems (25, 40).

For these forms of work-related problems, psychotherapy is the method of choice (10, 12, 30–32, 49, 52). Treatments need to be tailored to the work-related problem in focus. Thus, a thorough diagnostic assessment is the starting point for any treatment decision. This applies to both, a careful description on a phenomenological level (type of work-related problem) and on a level of functioning (maintaining mechanisms). Depending on the respective treatment approach, the latter may involve unresolved conflicts (55, 100), ego-functions (28, 29, 65) or dysfunctional cognitions (10) associated with the work-related problems. Psychodynamic approaches, for example, focus on the maintaining conflicts and ego-functions which also become manifest in the way someone “works” in therapy, e.g., in the way they handle the basic principle to speak as freely as possible (30). Thus, individuals may not adhere to the task, may not know where to start, may produce too many details, may not engage sufficiently and need constant encouragement or may tend to do all the therapeutic work themselves (in psychodynamic therapy, for example, patients interpreting the material themselves). An early example for a psychodynamic treatment of work-related problems is reported by Lang (101). According to Lang, Freud treated the famous conductor Bruno Walter who had developed an arm cramp that made conducting an orchestra impossible, by gradually encouraging him to move his arm while addressing the conductor's fear of disturbing the concert [(101), p. 93, (102), p. 234].

This exemplifies the idea that creative acts are changing our relationships with all sorts of others, in this case an audience (75). Drawing on Winnicott's idea that there is no such thing as an infant without a mother, there is also no such thing as a conductor without listeners or a writer without readers (75, 103). However, a real or imagined audience can be strict, critical, even brutal which may lead to negative reactions of the self and work-related problems (see above CCRT). Such relationship patterns may be understood and worked through in psychodynamic treatments with the goals of tolerating uncertainty, being playful, spontaneous, and without feeling the urge to adjust or correct original work to satisfy others.

Cognitive and cognitive-behavioral approaches include, for example, a functional analysis of work-related problems, an analysis of motivations and interpersonal aspects, a definition of goals and steps to reach them, task related interventions, working skills, identification and modification of problematic cognitions, improving interpersonal skills at the work-place, and measures to achieve a healthy work-life balance (10). Depending on the problem at hand, an exploration of certain basic work techniques should be undertaken. When these are not sufficiently available or developed, a positive treatment outcome is less likely. This entails prioritizing work related tasks and accomplishing tasks step by step in order to gain small successes and reduce anxiety, time management, practicing, doing unpleasant tasks first, finishing tasks without being distracted, finding a balance between leisure and work (10, 31).

As mentioned above, procrastination seems to be stronger when tasks are perceived as less enjoyable, i.e., “boring, frustrating, done resentfully, forced upon them by others and (…) generally more stressful, less meaningful and less structured” [(13), p. 165]. Thus, therapeutic or counseling approaches rooted in personality and motivational psychology argue for addressing these aversive aspects by finding projects more in line with core personal values and meaningful strivings (104, 105). See also strategies from acceptance and commitment therapy (Table 1).

Some more details on behavioral, cognitive-behavioral as well as third wave (acceptance and commitment therapy) and psychodynamic approaches to treatment can be found in Table 1.

For the treatment of perfectionism, a psychodynamic-interpersonal therapy has been developed which focuses on the dynamic and relational underpinnings of perfectionism (50). Perfectionism is regarded as both a defense against intolerable affects resulting from unfulfilled needs (e.g., for acceptance, self-worth) and as a means of gaining acceptance or garnering self-worth. However, the elusiveness of perfection implies the impossibility of having one's needs fulfilled (50). In psychodynamic-interpersonal therapy the interrelations of needs, affects, defenses and their relationship with significant others are worked through. The central aim is to help the patient accept that humanness includes strengths and limitations, all falling short of perfectionism [(50), p. 4].

With regard to empirical evidence, primarily various models of CBT with a focus on procrastination have been applied (12). With regard to self-help, there is evidence from an RCT including 150 participants that internet-based self-help using CBT principles is effective in reducing procrastination when compared to a waitlist control condition with moderate to large effect sizes for guided self-help and moderate effect sizes for unguided self-help (106). Clinically significant change was achieved among 31.3–40.0% of participants for guided self-help and 24.0–36.0% for unguided self-help. With regard to psychotherapy, CBT models proved to be superior to waiting list or no treatment (12, 107). In one study examining long-term outcome, results were stable at 1-year follow-up (108) with rates of 30–40% post-therapy and 8–36% at 1-year follow-up. However, the rates of patients achieving clinically significant improvements are limited (108, 109).

There is only anecdotal evidence or evidence from clinical experience for psychodynamic approaches to work-related problems, no evidence from randomized controlled trials (RCTs). For perfectionism, in a controlled but not randomized study short-term psychodynamic therapy proved to be superior to a waiting list in reducing perfectionism, depression and interpersonal problems with large effect sizes (49). Presently, a randomized controlled trial of psychodynamic treatment of perfectionism is being carried out (110).

There are no RCTs investigating the treatment of writer's block. However, treatment concepts have been proposed [e.g., (111)] and there are several published case illustrations focusing on psychoanalytic (83), behavioral (112) or paradoxical interventions (symptom prescription) (84).

A problem in RCTs targeting work-related problems is the primary endpoint. Studies addressing procrastination, for example, often assess a tendency for procrastination, but a reliable criterion for what constitutes a clinically meaningful amount of procrastination that justifies a need for professional treatment seems to be currently lacking (25).

## Future Research

For the reasons discussed above, there is a need to further improve the treatment of procrastination in particular and work-related problems in general and to offer individuals a variety of evidence-based treatments that may fit their treatment needs. To achieve these goals, the following approaches seem to be promising.

(1) With success rates of 30–40% one treatment does not seem to fit all. Thus, developing and applying further models of treatment is a promising approach.
(2) In the studies included in the meta-analysis by Malouff and Schutte (12) between 2 and 10 sessions were applied (107). Data on dose effect-relationships suggest that especially for chronic problems a higher treatment dose is required. By applying 20 or 24 sessions, success rates between 55 and 60% can generally be achieved (113).
(3) Furthermore, research has almost exclusively focused on procrastination, other types of work-related problems have been widely neglected. Treatments that address other forms of work-related problems need to be developed and tested empirically. They may be based on CBT, psychodynamic therapy, interpersonal therapy, systemic therapy or other models.

For example, for the various types of work-related problems described above a unified transdiagnostic and manual-guided psychodynamic treatment approach is presently being developed, integrating the existing psychodynamic treatment approaches (30–32, 49, 50, 114) in form of a unified protocol.

## Limitations

According to recent guidelines, there is no mandatory scheme on how to conduct a scoping review, however, certain methodological standards have been introduced to enhance the quality of this type of review (16, 17). In light of these standards, some limitations have to be mentioned. Firstly, we did not publish a pre-specified protocol. Secondly, we based our review on published findings in English and German which carries the risk of missing relevant aspects, especially with regard to cultural-specific phenomena. Thirdly, all authors have a psychodynamic background, which may have resulted in reporting bias. We tried to mitigate this potential effect by taking it critically into account while selecting topics, as well as by contacting two cognitive-behavioral scholars regarding further relevant literature. Fourthly, the present scoping review covers a wide range of topics in a field that is *per se* not well-defined, dates back many decades, but also has seen very recent developments. The iterative search and selection process, and the openness of the review team for a wide range of topics within the chosen field may have resulted in a broad and possibly not fully balanced review which can be seen as a first point of orientation toward further research. Finally, consumers and stakeholders were not included in designing and conducting the review.

## Conclusions

When Otto Kernberg was recently asked, how he would distinguish between an unconventional, eccentric or somehow odd person and a person with a personality disorder who needed therapy, he first answered by referring to the occupational functioning of that person: “…is he effective, on the top of his knowledge and his possibilities, is he satisfied with his work, does he get along well with his coworkers and so on.” [own translation, (115), p. 41]. Thus, according to Kernberg, whether an individual is able to work effectively, productively and satisfactorily may—among other things—be used to distinguish between normal and disordered personality and is considered essential in describing personality structure.

Considering the high prevalence of work-related problems with an even increasing tendency and associated psychosocial impairments, suffering, poor academic performance, more perceived stress and higher levels of depression and anxiety, they should become a more essential part of diagnostics in the mental health field. Psychotherapy is the method of choice to treat severe work-related problems (10, 12, 30–32, 49). At least for the treatment of procrastination, however, response rates are presently limited. For non-responders of the available treatments of procrastination, long-term treatments may be helpful and should be tested (113). No treatments have been proposed and tested for other types of work-related problems, with the exception of a psychodynamic treatment for perfectionism (49, 110). Thus, there is a need to further improve the treatment of work-related problems. For this purpose, the whole spectrum of work-related problems needs to be taken into account, not just procrastination. Further treatments based on various theoretical approaches need to be developed and validated.

## Author Contributions

CS and FL conceived of the idea of the article, performed literature searches, screened results, and wrote the first draft of the manuscript. NH critically revised the manuscript for intellectual content and aided in interpreting findings. All authors discussed the contents, methods and outline of the manuscript and approved the final version for publication.

## Dedication

This article is dedicated to Karl König who raised our interest in work-related problems.

## Conflict of Interest

The authors declare that the research was conducted in the absence of any commercial or financial relationships that could be construed as a potential conflict of interest.

## Publisher's Note

All claims expressed in this article are solely those of the authors and do not necessarily represent those of their affiliated organizations, or those of the publisher, the editors and the reviewers. Any product that may be evaluated in this article, or claim that may be made by its manufacturer, is not guaranteed or endorsed by the publisher.
